# TEX19 increases the levels of CDK4 and promotes breast cancer by disrupting SKP2-mediated CDK4 ubiquitination

**DOI:** 10.1186/s12935-024-03384-4

**Published:** 2024-06-12

**Authors:** Huantao Liu, He Wang, Hongyu Zhang, Miaomiao Yu, Yu Tang

**Affiliations:** 1 Department of Breast Surgery, Qilu Hospital of Shandong University, Jinan, China; 2 Department of Oncology, Cancer Hospital of China Medical University, Liaoning Cancer Hospital & Institute, Cancer Hospital of Dalian University of Technology, No.44 Xiaoheyan Road, Dadong District, Shenyang, Liaoning Province 110042 P. R. China

**Keywords:** Breast cancer, TEX19, CDK4, Ubiquitination

## Abstract

**Background:**

Globally, breast cancer in women is the fifth leading cause of cancer death. There is an urgent need to explore the molecular mechanism of breast cancer proliferation and metastasis.

**Method:**

TCGA database analysis was used to analyze genes expression in breast cancer and normal samples and the association between gene expression and prognosis. Immunohistochemical staining, qPCR and western blotting was sued to detected gene expression. The cell function tests were conducted to investigate the effects of TEX19 and CDK4 with abnormal expression on cell proliferation, migration, apoptosis, cell cycle, and colony formation. Bioinformatics analysis methods combined with CHX tracking experiment and Co-IP experiment were performed to screen and verify the downstream molecule and regulatory mechanism of TEX19. Besides, subcutaneous tumorigenesis model in nude mice was constructed.

**Results:**

TEX19 was significantly upregulated in breast cancer, and the TEX19 level was related to tumor invasion and prognosis. TEX19 knockdown inhibited the proliferation and migration of breast cancer cells, increased cell apoptosis, and blocked the cell cycle in the G2 phase. Besides, TEX19 suppressed the growth of tumors in the body. Mechanically, TEX19 upregulated the level of CDK4 protein, which depended on the E3 ubiquitin ligase SKP2. Specifically, TEX19 knockdown and SKP2 protein overexpression destroyed the stability of CDK4 protein and enhanced the ubiquitination of CDK4 protein. Additionally, CDK4 knockdown inhibited the proliferation, migration, and colony formation of breast cancer cells, and alleviated the promotion of TEX19 overexpression on the proliferation and migration of breast cancer cell.

**Conclusion:**

TEX19 and CDK4 were upregulated in breast cancer, and TEX19 increased the level of CDK4 protein by influencing SKP2-mediated ubiquitination of CDK4, thereby promoting the progression of breast cancer.

**Supplementary Information:**

The online version contains supplementary material available at 10.1186/s12935-024-03384-4.

## Background

Breast cancer in women has exceeded lung cancer as the leading cause of the global cancer incidence in 2020, and there are an estimated 2.3 million new cases. Globally, breast cancer in women is the fifth leading cause of cancer death, with 685,000 deaths [1]. The risk factors of breast cancer include genetic factors such as high-risk and intermediate-risk cancer susceptibility genes and single nucleotide polymorphisms associated with breast cancer, and non-genetic factors, such as personal history of breast disease, high mammographic density, exposure to therapeutic chest radiation, and the use of exogenous female hormones [2]. Despite the continuous advancement of diagnostic strategies, nearly 60% of breast cancer cases are still diagnosed at an advanced stage with a high mortality rate [3]. Local treatments for non-metastatic breast cancer include surgical resection and axillary lymph node sampling or resection, and postoperative radiotherapy is considered. Systemic therapy can be preoperative (neoadjuvant), postoperative (adjuvant) or both [4]. Nonetheless, it is reported that 20–30% of breast cancer patients may metastasize after diagnosis and treatment of primary tumors, and about 90% of cancer related deaths are attributable to metastasis. The 5-year overall survival rate of patients without metastatic breast cancer is more than 80%. However, distant metastasis can cause a sharp decrease in this rate to only 25% [5]. At present, almost all patients with metastatic breast cancer are still incurable. Systemic therapy of metastatic breast cancer is generally a new neoadjuvant/adjuvant method, and local treatments is generally surgery and radiation, which are usually only used to relieve symptoms and prolong patient life [4]. Consequently, it is urgent to explore the molecular mechanism of breast cancer proliferation and metastasis, in order to diagnose and treat breast cancer more effectively, thereby improving the survival and prognosis of breast cancer patients.

Testicular expression 19 (TEX19) is a confirmed cancer/testicular antigen (CTA), encoded by a mammalian-specific gene located at 17q25.3 of human chromosome 17 [6]. TEX19 is a mammalian orphan gene, that is, the encoded protein has no sequence similarity with any known protein [7]. However, in rodents, TEX19 was replicated to produce two paralogs, Tex19.1 and Tex19.2 which regulate the genomic stability and RNA expression of germline and placenta [8]. It has been proved that Tex19.1 is a nuclear factor that has a potential role in maintaining stem cells or pluripotency, besides, Tex19.1 also plays a vital role in meiosis and inhibition of retrotransposon [9]. Preliminary analysis of human TEX19 expression showed that TEX19 was homologous with Tex19.1 because it was expressed in human embryonic stem cells (ESC) [10]. Studies have reported that compared with low-grade bladder cancer, the expression of TEX19 in high-grade bladder cancer tissues is increased, which may play a role in the progression of bladder cancer [11]. Furthermore, the expression of TEX19 is associated with poor prognosis of certain cancers, and the reduction of TEX19 levels limits cell proliferation and growth of tumor volume, indicating that TEX19 is a necessary condition for tumor cell proliferation potential [12]. These results suggest that the expression of TEX19 can not only be used as a novel tumor biomarker, but also provide a tumor specific therapeutic target with broad-spectrum potential. However, the function of TEX19 in breast cancer and cancer cells is still unclear.

In this study, we analyzed the data of breast cancer patients in the TCGA database and found that the expression of TEX19 in breast cancer samples was significantly higher than that in normal samples, and there was a positively correlation between the expression level of TEX19 and Stage, T and N stages of breast cancer patients. The results of immunohistochemical staining of breast cancer tissue chips proved that TEX19 was indeed highly expressed in breast cancer tissues, and high levels of TEX19 indicated a poor prognosis, which suggested that TEX19 might play an important role in the progression and prognosis of breast cancer. We will further validate the role of TEX19 in breast cancer progression and explore possible molecular mechanisms to identify potential targets for the diagnosis and treatment of breast cancer, so as to improve the survival and prognosis of breast cancer patients.

## Methods

### Cell and tissue chip

Breast cancer cell lines (MDA-MB-231, MCF-7, MDA-MB-453 and BT549) and human normal mammary epithelial cell line (MCF-10 A) were purchased from BeNa, which were cultured in the DMEM medium supplemented with 10% FBS. All of the cells were cultured in the incubator containing 5% at 37 °C.

The tissue chip was purchased from Shanghai Outdo Biotech Company (China), containing 143 breast cancer tissues and 32 para-carcinoma tissues. Besides, the pathological characteristics of patients with breast cancer, such as age, grade, and tumor infiltrate, were collected and subjected statistical analysis.

### Bioinformatic analysis

GDC download tool was used to download the RNAseq counts document of breast cancer (BRCA) from TCGA database, containing 1097 breast cancer samples and 113 normal samples, and TCGAbiolinks package of R studio was used to download the clinical information of TCGA-BRCA patients. The estimate the dispersion in DEseq2 was used for data standardization, and the Benjamini-Hochberg (BH) was utilized to adjust P value. Differential analysis of gene expression was performed by DEseq2 and the screening criteria was as follows: |Fold Change| > 1.3 and *P* < 0.05.

The clinical information of breast cancer patients in TCGA database was from cbioportal (https://www.cbioportal.org/). The expression of TEX19 or CDK4 was divided into high expression group and low expression based on the optimal cut point, and then the difference of overall survival or progression-free survival between the two groups was analyzed by log rank test.

### Immunohistochemical staining (IHC)

Tissue samples were prepared into 5 μm tissue sections, and underwent antigen repair through citrate buffer solution. Tissue sections were blocked by 3% H_2_O_2_ and then by 5% serum. Tissue sections were incubated in the primary antibody solution at 4 °C overnight, and washed 3 times by 1 × PBST for 5 min each time. Tissue sections were incubated in the secondary antibody solution at 37 °C for 1 h and washed 3 times by 1 × PBST. DAB solution and tissue sections were incubated in the dark for 5 min, and the restained with hematoxylin (Baso) for 10–15 s. Tissue sections were washed by water and then separated by alcohol for 1–2 s. Finally, neutral gum was used for closed tissue sections, and the staining was observed under a microscope (Olympus) and photographed. IHC results were scored in terms of positive cells and staining intensity. IHC score = positive cell score × staining color intensity score. The higher the score, the higher the expression of protein to be detected. The relevant information of antibodies used in this experiment was provided in Supplementary Table 1.

### Lentivirus vector construction and cell infection

Construction of RNA interference lentivirus vector: Three RNAi target sequences were designed using TEX19 or CDK4 gene as templates, which were provided in Supplementary Table 2. RNAi sequences were synthesized single-stranded DNA oligo, and then annealed to form double-stranded DNA after being bathed at 90 °C for 15 min. Double-stranded DNA was attached to the linearized vector, and the ligated product was then transformed into the competent Escherichia coli cells. Subsequently, the plasmids were extracted according to the instructions for EndoFree Maxi Plasmid Kit (TIANGEN). The plasmids carrying the RNAi sequence were co-infected with the lentivirus packaging helper plasmids to 293T cells. ShTEX19 or shCDK4 lentivirus plasmids were extracted 72 h later.

Construction of overexpressed lentivirus vector: Using TEX19 gene as the template, the primer amplification sequence was designed. The synthesized primers were configured into PCR reaction system and PCR was carried out. The PCR products were connected to the linearized vector, and then co-transfected into the competent Escherichia coli cells. Subsequently, the plasmid extraction was performed using the EndoFree midi Plasmid Kit (TIANGEN). The plasmids carrying the TEX19 gene overexpression sequence were co-infected with the lentivirus packaging helper plasmids to 293T cells. TEX19 overexpression lentivirus plasmids were extracted 72 h later.

Cell infection by lentivirus: The infection solution was added to the culture dish of healthy growing breast cancer cells, along with the gene knockdown or overexpressed lentiviral plasmids (carrying green fluorescent protein), and then incubated in an incubator at 37 °C for 18 h. After the newly prepared medium was replaced, the culture was continued for 72 h, and the florescence was observed under a fluorescence microscope to evaluate the infection efficiency.

### Real time qPCR (qPCR)

Total RNA in breast cancer cells was abstracted by Trizol (Sigma) according to the kit operation instruction. cDNA was attained by reverse transcription of RNA under the instruction of Hiscripr QRT supermix for qPCR (+ gDNA WIPER) (Vazyme). The reaction system was prepared with SYBR Green mastermics (AceQ qPCR STBR Green master mix, Vazyme), forward and reverse primes, cDNA and other reagents in a certain proportion, and then Real Time PCR was performed in a two-step method on the Real Time PCR instrument (ABI), followed by the preparation of the dissolution curve. Formula 2^−△△Ct^ was used to calculate the mRNA levels of the gene. Primers used in qPCR assay were provided in Supplementary Table 3.

### Western blotting (WB)

After breast cancer cells were lysed, proteins in cell lysates were extracted. The protein concentration was determined with BCA Protein Assay Kit (HyClone-Pierce). 20 µg protein was suffered from SDS-PAGE, and then transferred to the PVDF membrane. PVDF membranes were incubated with TBST solution containing 5% skim milk at room temperature for 1 h, and then incubated with primary antibody solution at 4 °C overnight. PVDF membranes were cleaned by TBST for 3 times with 5 min each time. PVDF membranes and secondary antibody solution were co-incubated at room temperature for 1 h. After being washed by TBST, immobilon Western chemiluminescent HRP Substrote (Millipore) was utilized for color development of PVDF membranes and chemiluminescence was carried out using a Chemiluminescence get imaging system (GE). Antibodies used in western blotting assay were provided in Supplementary Table 1.

### Celigo cell counting assay

Breast cancer cells infected with lentivirus were digested with trypsin (Sangon Biotech (Shanghai) Co., Ltd), re-suspended into cell suspension, and inoculated into 96-well plate with 2000 cells per well. Starting the next day, Celigo (Nexcelom) scanned the target 96-well plate at the same point in time for 5 consecutive days to obtain the image. The scanned images were counted by Image J software and the cell proliferation curve was plotted.

### Flow cytometry

Flow cytometry was performed to detect cell apoptosis. When cell coverage in 6-well plate reached about 85%, breast cancer cells were re-suspended to cell suspension. After cleaning the cells with D-Hanks (pH = 7.2 ∼ 7.4) precooling at 4 °C, the cells were washed again with 1 × binding buffer. Cells were suspended with 200 µL 1 × binding buffer and incubated with 10 µL Annexin V-APC (eBioscience) at room temperature in the dark for 10–15 min. Afterwards, apoptosis levels were detected by flow cytometry (Millipore).

Flow cytometry was conducted to determine cell cycle. When the cells in each group grew to about 80% coverage, the cells were digested with trypsinase and collected in a 5 mL centrifuge tube. The cells were washed with PBS (pH = 7.2 ∼ 7.4) precooling at 4 °C for one time, and fixed in 70% ethanol precooling at 4 °C for at least 1 h. After washing the cells with PBS, the cells were re-suspended with the prepared cell staining solution, and the cell cycle distribution was detected by flow cytometry (Millipore). The proportion of cell staining solution was as follows: 40 × PI solution (2 mg/mL) (Sigma):100 × RNase solution (10 mg/mL):1 × PBS = 25:10:1000.

### Human apoptosis antibody array

Human Apoptosis Antibody Array (ab134001) was purchased from abcam, with a total of 43 apoptosis markers. According to the instruction, Human Apoptosis Antibody Array could simultaneously detect the concentration of 43 apoptosis markers in MDA-MB-231 cells of the TEX19 knockdown group and the shCtrl group. Total proteins were extracted from MDA-MB-231 cells infected with shTEX19 or shCtrl lentivirus after lysis. The protein concentration was determined with BCA Protein Assay Kit. The membrane was incubated with 2 mL 1 × Blocking buffer at room temperature for 30 min, and then incubated with 1.2 mL samples at 4 °C overnight. After that, the membrane was incubated in 1 mL of 1 × Biotin-conjugated Anti-Cytokines and 1.5 mL of 1 × Streptavidin-HRP successively. Finally, protein levels were detected under the instruction of Chemiluminescent Detection protocol.

### Transwell assay

The Transwell upper chamber (3422, corning) was incubated with 100 µL serum-free medium for 2 h. The medium in upper chamber was replaced with 100 µL cell suspension (containing 50,000 breast cancer cells). Afterwards, the upper chamber was transferred to the lower chamber containing 600 µL medium with 30% FBS for incubation for 24 h. The medium was removed from the upper chamber and the non-metastatic cells were gently swabbed with a cotton swab. After that, the upper chamber was soaked in 400 µL Gimsa solution for 5 min. The film was rinsed with water and dried in air. Finally, the cell metastasis was observed under a microscope (Olympus) and photographed.

### Wound healing

Lentivirus-infected breast cancer cell suspensions were added to 96-well plate, 40,000 cells per well. The next day, the 96-well plates were gently pushed upward from the lower central part with 96 Wounding Replicator (VP scientific), and rinsed 3 times with serum-free medium. Medium with 0.5% FBS was added and cells were cultured in an incubator containing 5% CO_2_ at 37 °C. The plates were scanned with Cellomics (Thermo) at the right time according to the degree of healing. The scanning time of MDA-MB-231 cells was 0 h, 8 h, and 24 h. The scanning time of BT549 cells was 0 h, 24 h, and 48 h. Cellomics was employed to evaluate cell migration by analyzing cell area from the same field of view at different time points.

### Subcutaneous tumorigenesis model in nude mice

4-week-old BALB/c nude mice (female) were purchased from Beijing Vital River Laboratory Animal Technology Co., Ltd. Nude mice were randomly divided into control group (shRNA group) and experimental group (shTEX19 group) with 10 nude mice in each group. 200 µL cell suspension (4 × 10^6^ MDA-MB-231 cells infected with shTEX19 or shRNA) was injected subcutaneously into nude mice. From day 25 onwards, the long and short diameter of the tumors were measured every 10 days with a Vernier caliper, and the tumor volume was calculated to plot the tumor growth curve. Tumor volume: V = π/6 × L × W × W. V represented tumor volume; L represented the long diameter for the tumor; W represented the short diameter of the tumor. On the last day of the experiment, 0.7% sodium pentobarbital (10 µL/g) (SIGMA) was injected intraperitoneally, and then anesthetized nude mice were imaged using the small animal living imaging system (BERTHOLD TECHOLOGIES). Fluorescence was observed and photographs were taken. The tumors were removed from the sacrificed nude mice, weighed and measured in volume, and then photographed with a digital camera (SONY). Immunohistochemical staining was used to detect the expression of Ki67 protein in tumor tissues, as described above. The antibodies used in this experiment were provided in Supplementary Table 1.

### GeneChip and ingenuity pathway analysis (IPA)

Total RNA in breast cancer cells (MDA-MB-231) was abstracted by Trizol (Sigma) according to the kit operation instruction. According to the instruction manual of Affymetrix’s gene chip sequencing instrument, the whole gene expression profile chip experiment based on GeneChip was performed on the total RNA. The original chip data was preprocessed including KNN function missing value filling, data normalization, and data cleaning. In the process of hierarchical clustering significance difference analysis of preprocessed data using R studio, a linear model based on empirical Bayesian distribution was used to calculate the P-value of significant difference level, and Benjamini-Hochberg method was employed to correct the significant difference level (FDR). Significant difference of gene screening standard was: |Fold Change| ≥ 1.3 and P-value < 0.05. Based on the differentially expressed genes, the classical pathway enrichment analysis and molecular interaction network analysis were performed using IPA.

### Co-immunoprecipitation (Co-IP)

After breast cancer cells (MDA-MB-231 and BT549) were lysed, proteins in cell lysates were extracted. The protein concentration was determined with BCA Protein Assay Kit. The lysate containing 1.2 mL total protein and the reference antibody were incubated at 4 °C overnight under rotating conditions. Then the incubation product and 200 µL beads were incubated at 4 °C for 2 h under rotating conditions, followed by centrifugation at 2000 × g centrifugal force for 1 min. The IP product was cleaned 2 times with IP lysate and subjected SDS-PAGE. The proteins were transferred to the PVDF membrane. PVDF membranes were incubated with TBST solution containing 5% skim milk at room temperature for 1 h, and then incubated with primary antibody solution at 4 °C overnight. PVDF membranes were cleaned by TBST for 3 times with 5 min each time. PVDF membranes and secondary antibody solution were co-incubated at room temperature for 2 h, and washed by TBST. Chemiluminescence was carried out using a Chemiluminescence get imaging system. Antibodies used in western blotting assay were provided in Supplementary Table 1.

### Protein stability assay

Cycloheximide (CHX, 0.2 mg/mL, S7418, Selleck), an inhibitor of protein synthesis, was used to treat MDA-MB-231 and BT549 cells with TEX19 knockdown or SKP2 overexpression to evaluate protein stability. After 0, 3, 6, and 12 h of CHX treatment of cells, total proteins in MDA-MB-231 cells were extracted. Then 20 µg total protein was subjected to western blotting to detect CDK4 protein levels. Information about relevant primary and secondary antibodies was provided in Supplementary Table 1.

### Ubiquitination assay

MDA-MB-231 and BT549 cells were infected with shTEX19 or SKP2 overexpression lentivirus for 24 h, and incubated with ubiquitin-proteasome pathway inhibitor MG-132 (20 µM, HY-13,259, MEC) for 6 h. Total proteins in MDA-MB-231 cells were extracted. 20 µg total protein was subjected to western blotting to detect CDK4 protein levels. Another1.0 mg total protein and antibody were incubated at 4 °C overnight, and then incubated with 20 µL beads at 4 °C for 2 h. The protein-antibody-beads complex was subjected to western blotting, and the levels of ubiquitin were determined by the ubiquitin antibody. Information about relevant primary and secondary antibodies was provided in Supplementary Table 1.

### CCK8 assay

MDA-MB-231 and BT 549 cells in the logarithmic growth phase were suspended and added to the 96-well plate at 100 µL per well (2000 cells). At the 1st, 2nd, 3rd, 4th and 5th day, one 96-well plate was removed for CCK8 detection. Specifically, 10 µL CCK8 reagent (Sigma) was added to each well and incubated with cells for 4 h. After the 96-well plates were oscillated for 2 min, the OD values were detected at 450 nm wavelength by using a microplate reader (M2009PR, Tecan infinite), and the fold change of OD value was calculated.

### Colony formation assay

MDA-MB-231 and BT549 cells were inoculated in 6-well plates with 2 mL cell suspension per well, containing 1000 cells. The cells were cultured for 8 days, during which the medium was changed every 3 days. 1 mL 4% paraformaldehyde was added per well to fix cells for 30–60 min. The cells were washed once with PBS and incubated with 500 µL GIEMSA solution (Shanghai Dingguo Biological Technology Co., Ltd.) for 10–20 min. After cleaned several times with ddH2O, a digital camera was used to take pictures and the clones was counted.

### Statistical analysis

Sign test were employed to evaluate whether TEX19 gene expression was statistically different between breast cancer tissues and para-carcinoma tissues. Chi-square Test and Mann-Whitney U analysis were used to analyze the signification of TEX19 expression at different levels in different pathological characteristics, and Spearman correlation analysis was conducted to analyze the correlation between the expression level of TEX19 in cancer tissues and pathological characteristics. T-test was used for statistical analysis between the other two groups, and One-way ANOVA was utilized for statistical analysis among multiple groups. *P* < 0.05 represented significant difference.

## Results

### TEX19 had a high expression in breast cancer and associated with tumor infiltrate

**Fig. 1:**
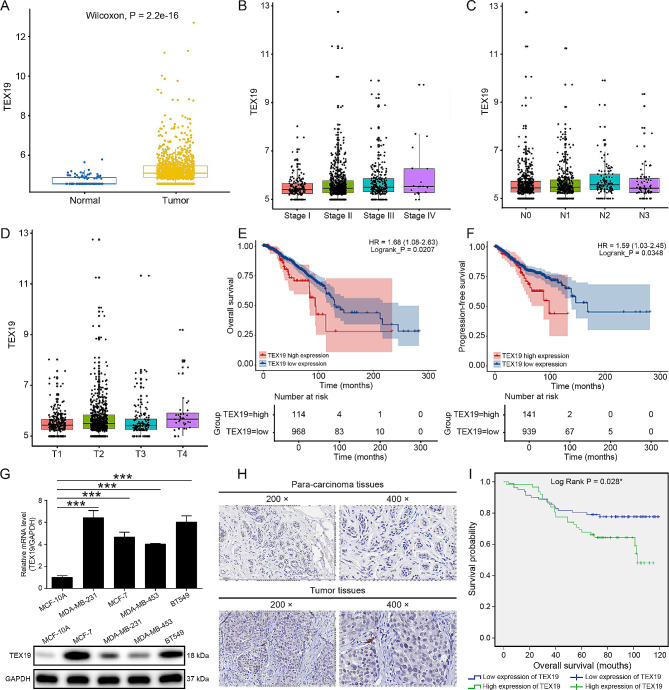
TEX19 was highly expressed in breast cancer. (**A**) The expression of TEX19 in the TCGA database was significantly higher in breast cancer than that in normal tissues. (**B**) The expression of TEX19 in the TCGA database was markedly different among different tumor stages of breast cancer patients. (**C**) The expression of TEX19 in the TCGA database was obviously different among different N grades of breast cancer. (**D**) The expression of TEX19 in the TCGA database was obviously different among different T grades of breast cancer. (**E**) The expression of TEX19 was divided into high expression group and low expression based on the optimal cut point, and then the difference of overall survival between the two groups was tested by log rank test. (**F**) The difference of progression-free survival between the high expression group of TEX19 or the low expression group of TEX19 was tested by log rank test. (**G**) qPCR and western blotting were performed to detect the mRNA and protein levels of TEX19 in MCF-10 A and breast cancer cell lines. (**H**) Immunohistochemical staining was carried out to assess the levels of TEX19 protein in breast cancer tissues and para-carcinoma tissues. The magnification: 200 × and 400 ×. (**I**) Kaplan-Meier analysis was used to evaluate the association between TEX19 levels and prognosis in breast cancer patients. *P < 0.05, ***P < 0.001

According to the TCGA database, the expression of TEX19 in breast cancer tissues was higher than that in normal tissues (Fig. 1A), and was significantly different in tumor tissues of patients with different Stage, T and N stages. Further Spearman correlation analysis indicated that the levels of TEX19 in tumor tissues was positively correlated with Stage, T and N stages of patients, suggesting that the levels of TEX19 gene could be used as an indicator for clinical diagnosis of Stage, T and N stages of patients (Fig. 1B and D). Besides, the analysis results of log rank test indicated that high expression of TEX19 predicted a poor overall survival (Fig. 1E) and progression-free survival (Fig. 1F). Compared to normal breast epithelial cells (MCF-10 A cells), the mRNA and protein levels of TEX19 were upregulated in breast cancer cell lines (MDA-MB-231, MCF-7, MDA-MB-453 and BT549 cell lines) (Fig. 1G). Resembled results were obtained based on the immunohistochemical staining results of the tissue chip, with higher levels of TEX19 protein in breast cancer tissues compared with para-carcinoma tissues (Fig. 1H; Table 1). Additionally, according to Spearman correlation analysis, the expression of TEX19 was positively correlated with Tumor Infiltrate and Tumor size (Tables 2 and 3). Moreover, high expression of TEX19 gene was significantly associated with shorter overall survival of breast cancer according to Kaplan-Meier analysis (Fig. 1I). These results suggested that TEX19 may play a vital role in the progression of breast cancer.

**Table 1:** The expression of TEX19 in breast cancer tissues and para-carcinoma tissues by immunohistochemistry analysis

TEX19 expression	Tumor tissue		Para-carcinoma tissue		p value
	Cases	Percentage	Cases	Percentage	
Low	81	56.6%	32	100%	< 0.001***
High	62	43.4%	0	–	

**Table 2:** Relationship between TEX19 expression and pathological characteristics in patients with breast cancer

Features	No. of patients	TEX19 expression		*p* value
		low	high	
All patients	143	81	62	
Age (years)				0.143
< 58	70	44	26	
≥ 58	73	37	36	
Grade				0.378
I	1	0	1	
II	71	44	27	
III	61	32	29	
AJCC stage				0.239
1	24	15	9	
2	77	46	31	
3	37	18	19	
Tumor Infiltrate				0.039*
1	36	26	10	
2	88	46	42	
3	13	5	8	
4	2	2	0	
lymphatic metastasis (N)				0.480
0	74	43	31	
1	36	22	14	
2	19	10	9	
3	12	5	7	
Tumor size				0.026*
< 3 cm	59	40	19	
≥ 3 cm	80	39	41	
Lymph node positive				0.822
= 0	70	40	30	
> 0	67	37	30	

**Table 3:** Correlation analysis of TEX19 gene expression with tumor Infiltrate or tumor size in patients with breast cancer

		TEX19
Tumor Infiltrate	Spearman correlation	0.176
	Significance (two-tailed)	0.039*
	N	139
Tumor size	Spearman correlation	0.190
	Significance (two-tailed)	0.025*
	N	139

### TEX19 knockdown inhibited the proliferation of breast cancer cells and suppressed tumor growth

**Fig. 2:**
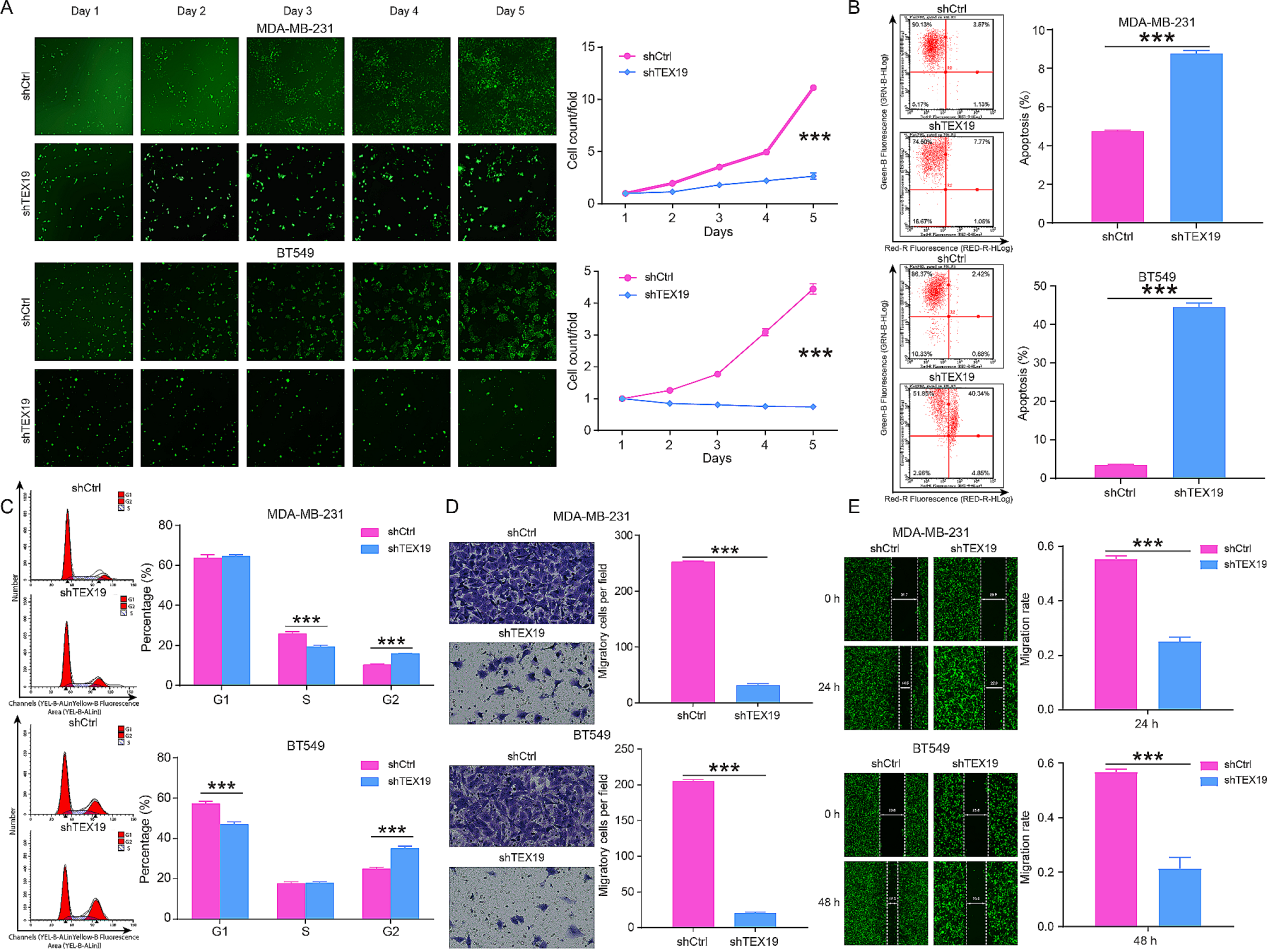
TEX19 knockdown inhibited proliferation and migration but induced apoptosis of MDA-MB-231 and BT549 cells. (**A**) The proliferation of MDA-MB-231 and BT549 cells was detected by Celigo cell counting assay after TEX19 knockdown, and it was found that TEX19 knockdown slowed down the cell proliferation rate. (**B**) Flow cytometry was used to determine the apoptosis of MDA-MB-231 and BT549 cells after TEX19 silence. (**C**) The effect of shTEX19 on cell cycle MDA-MB-231 and BT549 cells was demonstrated using Flow cytometry. (**D**) Transwell was performed to evaluate the migration ability of MDA-MB-231 and BT549 cells after TEX19 silence. (**E**) Wound healing was performed to evaluate the migration ability of MDA-MB-231 and BT549 cells after TEX19 silence. **P < 0.01, ***P < 0.001. These cell assays were repeated for 3 times

**Fig. 3:**
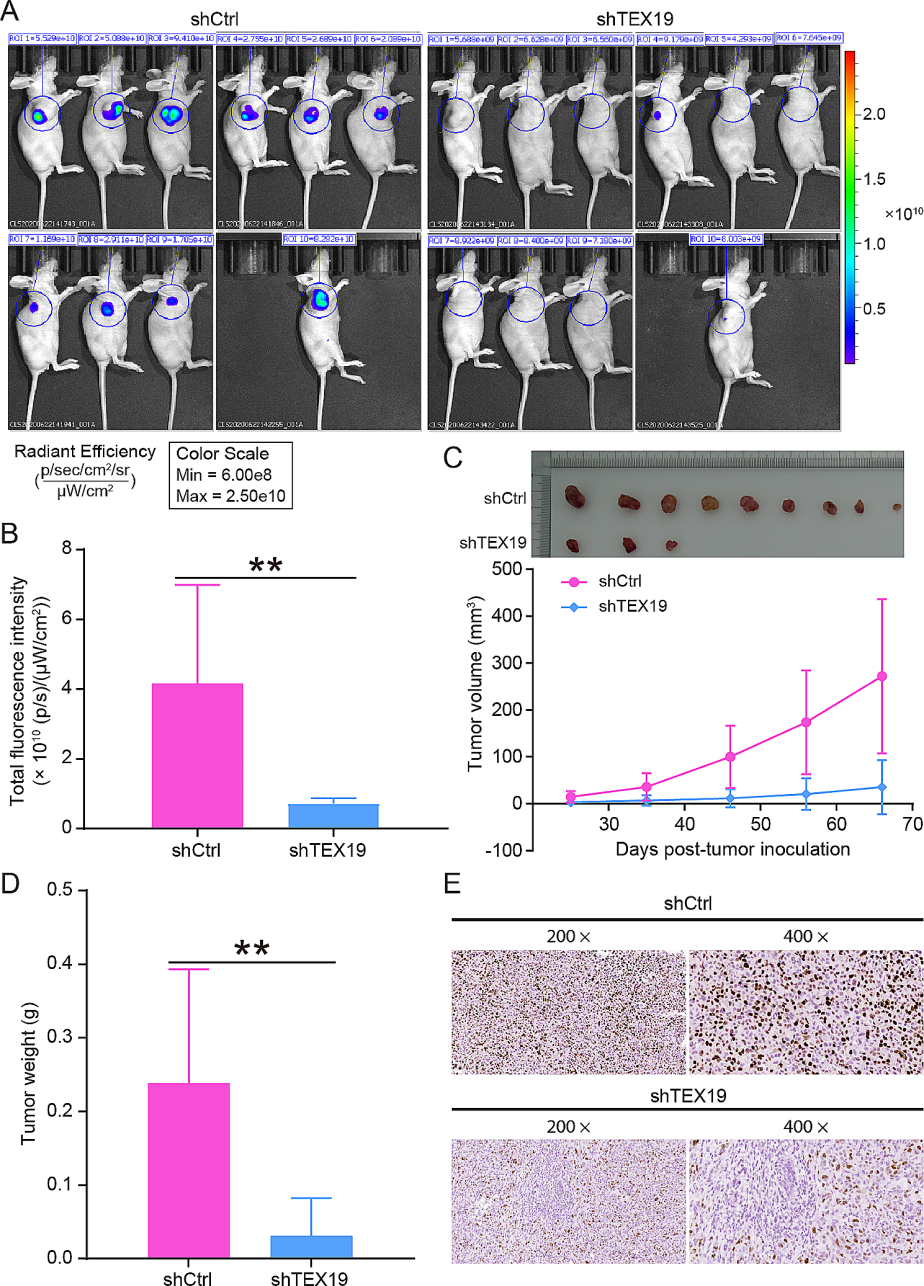
TEX19 downregulation suppressed the growth of tumors in vivo. (**A**-**B**) In vivo imaging showed the fluorescence intensity of tumors in nude mice, and the effects of TEX19 downregulation on tumor growth in vivo was analyzed. (**C**) Tumor volume growth curves in nude mice of shCtrl group and shTEX19 group. (**D**) Tumors taken from nude mice were weighted and found to be significantly lighter in the shTEX19 group compared with the shCtrl group. (**E**) The expression of Ki67 protein in solid tumor tissues in the shCtrl and shTEX19 groups was determined to assess the proliferation ability of tumor cells. The magnification: 200 × and 400 ×. **P < 0.01

RNA interference sequences were designed using TEX19 as the template, and shTEX19-1 with better knockdown effect was selected to continue the subsequent experiments (Fig. S1A). Fluorescence observation, qPCR and western blotting results showed that the infection efficiency of breast cancer cells (MDA-MB-231 and BT549) reached more than 85%, and shTEX19 lentivirus could markedly decline the levels of TEX19 mRNA and protein, suggesting that TEX19 knockdown models of breast cancer cells were successfully constructed (Fig. S1B–D). At the levels of cell function, downregulation of TEX19 inhibited the proliferation of breast cancer cells MDA-MB-231 and BT549 (Fig. 2A), heightened cell apoptosis (Fig. 2B), enhanced the proportion of G2 phase (Fig. 2C), and restrained cell migration (Fig. 2D and E). Furthermore, the results of Human Apoptosis Antibody Array revealed that the silence of TEX19 augmented the expression of apoptosis pathway related proteins BIM, Caspase3, Caspase9, Fas, HTRA, IGFBP-5, p27, p53, and SMAC, and refrained the expression of IGF-II, Survivin and XIAP (Fig. S2A-B). MDA-MB-231 cells infected with shTEX19 lentivirus were subcutaneously injected into nude mice. Comparison with shCtrl group, tumor growth was slower and Ki67 expression was lower in the shTEX19 group, suggesting that downregulation of TEX19 suppressed tumor growth and proliferation in vivo (Fig. 3A and E).

#### TEX19 regulated the levels of CDK4 through SKP2-mediated ubiquitination

**Fig. 4:**
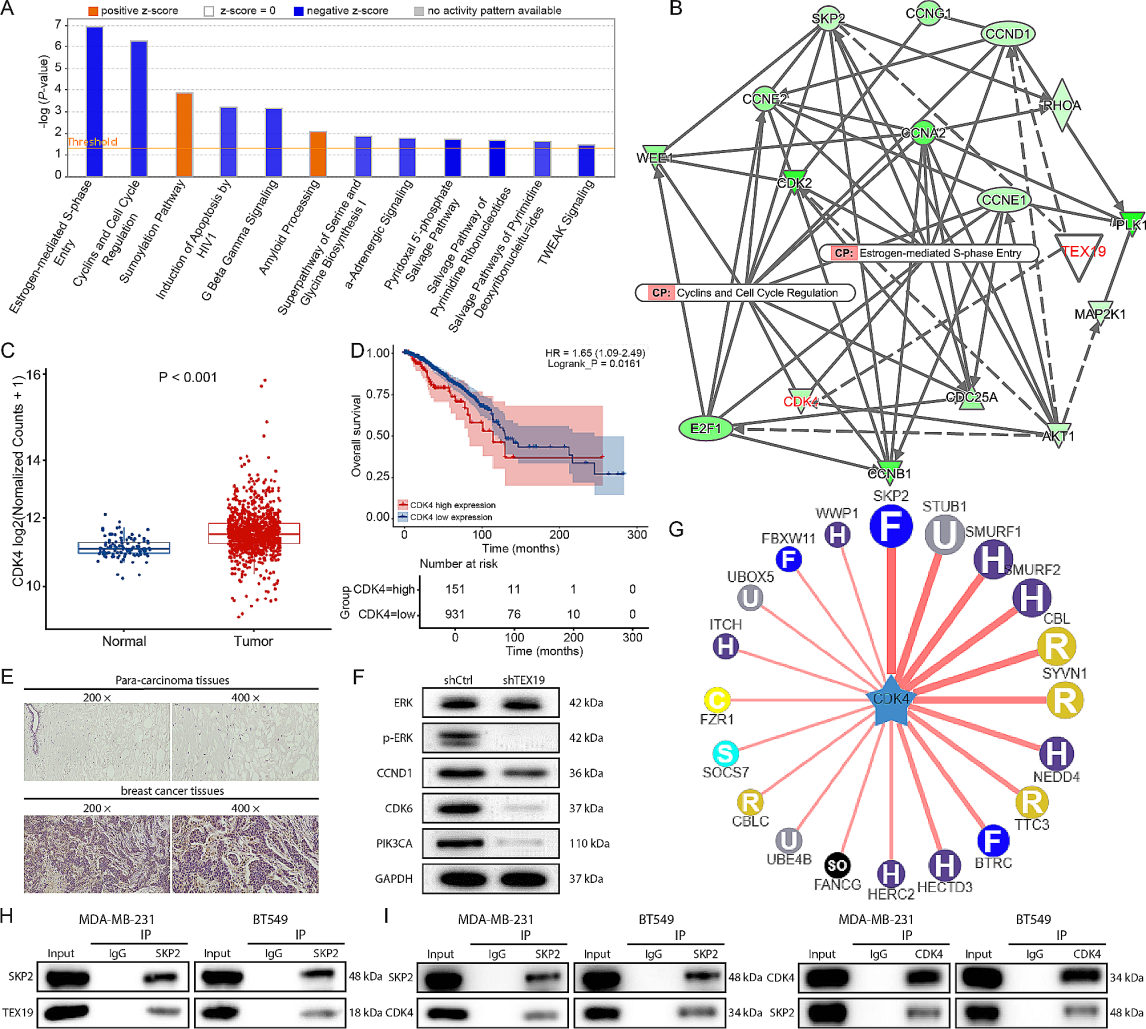
TEX19 regulated CDK4 protein levels by interacting with SKP2. (**A**) IPA was used to conduct enrichment analysis of classical pathways to explore the enrichment of differential genes in classical pathways. (**B**) The molecular interaction network of TEX19, CDK4 and the classical pathways Cyclins and Cell Cycle Regulation, Estrogen-mediated S-phase Entry related proteins. (**C**) The expression of CDK4 in the TCGA database was significantly higher in breast cancer than that in normal tissues. (**D**) The difference of overall survival between the high expression group of CDK4 or the low expression group of CDK4 was tested by log rank test. (**E**) Immunohistochemical staining was carried out to assess the levels of CDK4 protein in breast cancer tissues and para-carcinoma tissues. The magnification: 200 × and 400 ×. (**F**) western blotting was conducted to detect the expression of related protein in the pathways (ERK, p-ERK, CCND1, CDK6, PIK3CA) in the MDA-MB-231 cells infected with shTEX19 lentivirus. (**G**) The ubiquitin ligase (E3) acting on CDK4 was predicted by UbiBrowser. (**H**) Co-IP assay was used to demonstrate the interaction between TEX19 and SKP2. (**I**) Co-IP assay was conducted to confirm the interaction between SKP2 and CDK4

GeneChip was conducted on MDA-MB-231 cells in the TEX19 knockdown group and the control group, the results of which were subjected to hierarchical clustering significance (Fig. S3A). Several significantly differentially expressed genes were selected to verify the expression of their mRNA and proteins in TEX19 knockdown cells. The results showed that TEX19 knockdown not only dramatically reduced CDK4 mRNA levels, but also obviously inhibited CDK4 protein expression (Fig. S3B-C). The enrichment analysis of classical pathway by IPA suggested that Estrogen-mediated S-phase Entry and Cyclins and Cell Cycle Regulation were inhibited in TEX19 knockdown breast cancer cells (Fig. 4A). Further, there were interactions among TEX19, CDK4, and some related proteins in the Estrogen-mediated S-phase Entry and Cyclins and Cell Cycle Regulation pathways (Fig. 4B). According to the TCGA database, CDK4 was highly expressed in breast cancer tissues compared to the normal tissues (Fig. 4C), and the high expression of CDK4 was negatively associated with the prognosis of breast cancer patients (Fig. 4D). Subsequent assays indicated that CDK4 was highly expressed in breast cancer tissues (Fig. 4E). shTEX19 inhibited the phosphorylation of ERK protein, and the expression of CCND1, CDK6 and PIK3CA proteins in breast cancer cells (Fig. 4F). These results determined that TEX19 might play a vital role in breast cancer cells by regulating CDK4 expression and Estrogen-mediated S-phase Entry and Cyclins and Cell Cycle Regulation pathways.

**Fig. 5:**
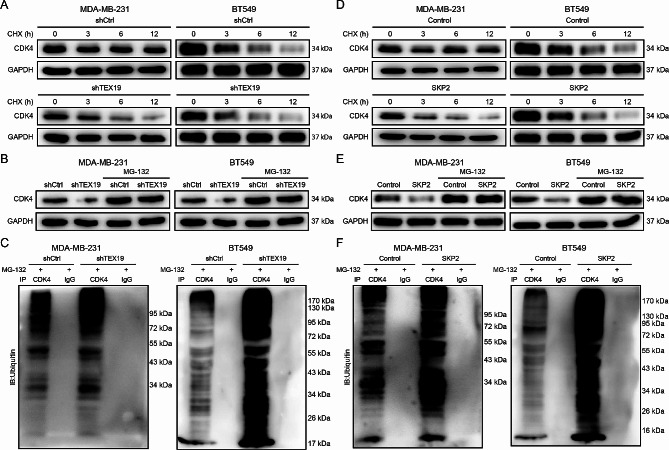
TEX19 regulated the SKP2-involved ubiquitination of CDK4. (**A**) The MDA-MB-231 and BT549 cells were treated with or without knockdown of TEX19 with a protein synthesis inhibitor CHX, and then the levels of CDK4 protein were detected by western blotting after 0, 3, 6 and 12 h. (**B**) The proteasome inhibitor MG-132 was used to treat TEX19 knockdown MDA-MB-231 and BT549 cells, and the levels of CDK4 protein were detected by western blotting. (**C**) The TEX19 knockdown MDA-MB-231 and BT549 cells were treated with MG-132, and then the levels of CDK4 ubiquitination were assessed by Co-IP assay. (**D**) The breast cancer cells were treated with or without overexpression of SKP2 with CHX, and then the levels of CDK4 protein were detected by western blotting after 0, 3, 6 and 12 h. (**E**) MG-132 was treated SKP2 overexpression MDA-MB-231 and BT549 cells, and the levels of CDK4 protein were detected by western blotting. (**F**) The SKP2 overexpression breast cancer cells were treated with MG-132, and then the levels of CDK4 ubiquitination were assessed by Co-IP assay

Post-translational modification (PTM) has been shown to affect protein structure and function, and the ubiquitin-proteasome system (UPS) is responsible for the degradation of most proteins in eukaryotic cells [13, 14]. UbiBrowser database predicted that the E3 ubiquitin ligase SKP2 and CDK4 had a strong relationship (Fig. 4G), and previous reports showed that SKP2 interacted with CDK4 and enhanced the ubiquitination and degradation of CDK4 [15]. Besides, Co-IP assay showed an interaction between TEX19 and SKP2 proteins (Fig. 4H), as well as between SKP2 and CDK4 (Fig. 4I). Therefore, we suspected that TEX19 might regulate the level of CDK4 protein through ubiquitin-proteasome. When CHX was used to inhibit protein synthesis in MDA-MB-231 and CDK4 cells, the degradation of CDK4 protein was accelerated through TEX19 knockdown (Fig. 5A). MG-132 treatment of MDA-MB-231 and CDK4 cells with TEX19 knockdown restored the inhibition of CDK4 protein expression by TEX19 knockdown or SKP2 overexpression (Fig. 5B). Additionally, TEX19 knockdown significantly increased the ubiquitin level of CDK4 protein (Fig. 5C). Similar to the results induced by TEX19 knockdown, SKP2 overexpression disrupted the stability of CDK4 protein (Fig. 5D), which depended on the proteasome degradation pathway (Fig. 5E). SKP2 overexpression significantly increased the ubiquitin level of CDK4 protein (Fig. 5F). The above results proved that TEX19 inhibited the ubiquitination of CDK4 mediated by SKP2 and thus enhanced the levels of CDK4 protein.

#### TEX19 promoted the progression of breast cancer by regulating CDK4

RNA interference sequences were designed using CDK4 as the template, and shCDK4-1 with better knockdown effect was selected to continue the subsequent experiments (Fig. S4A). The results of qPCR and western blotting indicated that TEX19 was markedly upregulated in the TEX19 overexpression group and shCDK4 dramatically cut down the levels of CDK4 mRNA and protein (Fig. S4B-C). These results suggested that TEX19 overexpression, CDK4 knockdown, and CDK4 knockdown with TEX19 overexpression models of breast cancer cells were successfully constructed.

**Fig. 6:**
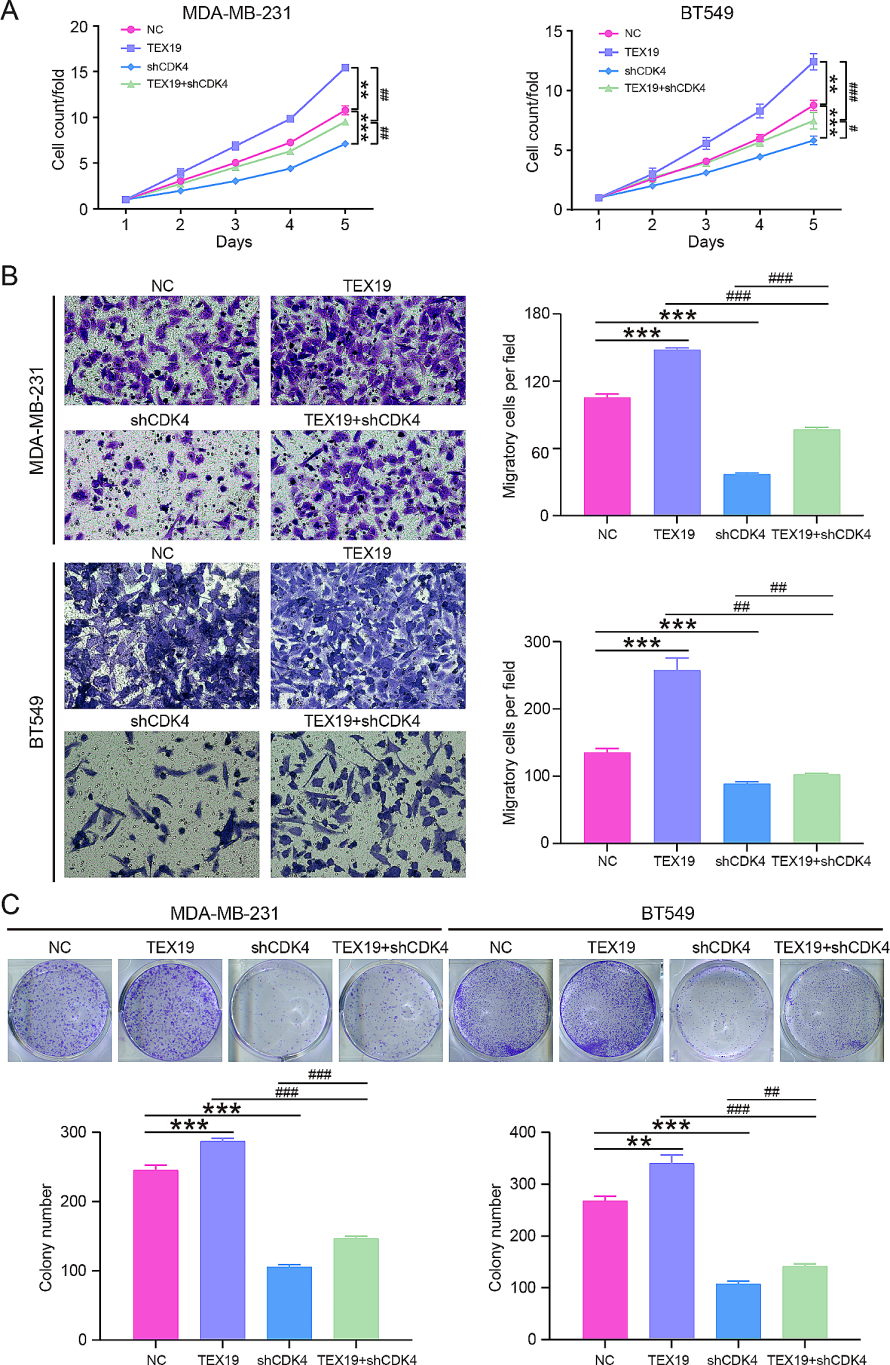
TEX19 prompted the progression of breast cancer by interacting with CDK4. (**A**) The proliferation of MDA-MB-231 and BT549 cells was detected by CCK8 assay after genes overexpression or knockdown. (**B**) Transwell was performed to evaluate the migration ability of MDA-MB-231 and BT549 cells after genes overexpression or silence. (**C**) Colony formation assay was conducted to analyze the colony formation ability of MDA-MB-231 and BT549 cells after genes overexpression or silence. **P < 0.01, ***P < 0.001, compared to the NC group. #*P* < 0.05, ##*P* < 0.01, ###*P* < 0.001, compared to the TEX19 or shCDK4 group. These cell assays were repeated for 3 times

At the levels of cell function, TEX19 overexpression inhibited the proliferation of MDA-MB-23 and BT549 cells, while CDK4 knockdown showed the opposite effect, that is, shCDK4 restricted the proliferation of MDA-MB-23 and BT549 cells. In addition, downregulation of CDK4 partially restored the enhanced cell proliferation caused by TEX19 upregulation (Fig. 6A). TEX19 overexpression and CDK4 silence had opposite effects on the migration of MDA-MB-23 and BT549 cells, and the promotion effect of TEX19 overexpression was neutralized by the blocking effect of CDK4 silence (Fig. 6B). Furthermore, the colony formation capacity of MDA-MB-231 and BT549 cells was enhanced in the TEX19 overexpression group and restricted in the shCDK4 group, and the downregulation of CDK4 restrained the increase of colony formation capacity inducted by the upregulation of TEX19 (Fig. 6C). All of these results revealed that TEX19 promoted the progression of breast cancer by upregulating CDK4.

## Discussion

Breast cancer is the most common and deadliest cancer affecting women worldwide [16]. Although postoperative comprehensive treatment has been effective in prolonging survival, 10–15% of breast cancer patients still have disease progression and distant metastasis within 3 years after initial diagnosis [17]. To further improve breast cancer outcomes, it is urgent to explore the molecular mechanisms of breast cancer progression and metastasis. In our research, TEX19 was significantly highly expressed in breast cancer, and the levels of TEX19 protein were positively correlated with the degree of tumor infiltration. In addition, patients with higher TEX19 levels had shorter survival times and progression-free survival. These results indicated that TEX19 might play an important role in the progression and prognosis of breast cancer.

Tumor cells have many characteristics, such as self-proliferation ability, anti-apoptosis, unlimited replication potential, tissue invasion and metastasis [18]. TEX19 protein has been reported as a cancer/testicular antigen, which may be a potential therapeutic target for cancer treatment [19, 20]. Studies have shown that TEX19 is highly expressed in ovarian cancer, and its knockdown restricts the proliferation, migration and invasion of ovarian cancer cells, indicating that TEX19 has a cancer-promoting effect in ovarian cancer [6]. In our research, knockdown of TEX19 significantly inhibited the proliferation and migration of breast cancer cells, induced apoptosis, and blocked cells in the G2 phase of the cell cycle. The opposite results were obtained after overexpression of TEX19, that is, TEX19 overexpression promoted cell proliferation and migration and inhibited apoptosis. In addition, knockdown of TEX19 also suppressed tumor growth in vivo. These suggested that TEX19 promoted the progression of breast cancer.

In order to investigate the molecular mechanism of TEX19 in breast cancer, we found an indirect interaction between TEX19 and CDK4 through bioinformatics analysis and IPA. The expression levels of CDK4 in breast cancer tissues were significantly higher than that in para-carcinoma tissues, and TEX19 upregulated the expression of CDK4. CDK4 is a member of the cyclin-dependent kinase (CDK) family. CDK is a hallmark of cancer and is involved in the regulation of processes such as proliferation, migration, apoptosis and angiogenesis [21]. However, the specific mechanism of TEX19 regulating CDK4 protein was not clear. Previous studies have shown that UPS affects a variety of cell functions, such as cell proliferation, cell cycle progression, transcription and cell apoptosis, mainly by regulating the stability of proteins [22, 23]. Tex19.1 can regulate the activity of the E3 ubiquitin ligase UBR2 protein, and then play a role in the female germline [24]. Human TEX19 promotes ubiquitin-dependent degradation of downstream protein LINE-1 by acting on UBR2 [25]. Besides, the E3 ubiquitin ligase SKP2 enhances the ubiquitination and degradation of CDK4 by acting on the C-terminal lobe of CDK4 [15]. Therefore, we hypothesized that TEX19 regulated the ubiquitination and degradation of CDK4 protein by acting on E3 ligase. The prediction results of UbiBrowser database revealed that SKP2 and CDK4 did have a strong relationship, and there was an interaction relationship between TEX19 and SKP2 proteins as well as SKP2 and CDK4 proteins. SKP2 is expressed in many tissues and participates in a variety of cell functions, such as cell proliferation, metabolism, and tumorigenesis by promoting the ubiquitination and degradation of a variety of tumor-related genes [26], and overexpression of SKP2 was negatively correlated with overall survival of breast cancer patients [27]. TEX19 knockdown or SKP2 overexpression accelerated the degradation of CDK4 protein, and MG-132 restored the downregulation of TEX19 knockdown or SKP2 overexpression on the level of CDK4 protein. Furthermore, knockdown of TEX19 and overexpression of SKP2 significantly increased the ubiquitin level of CDK4 protein. The above results testified that TEX19 inhibited the ubiquitination of CDK4 mediated by SKP2, thereby enhancing the expression of CDK4 protein.

CDK4/6 are fundamental drivers of the cell cycle and are necessary for the occurrence and development of various malignant tumors [28]. CDK4/6 specifically regulates cell transition from G1 phase to S phase of the cell cycle. CDK4/6 inhibitors effectively block the proliferation of sensitive cancer cells by inducing G1 cell cycle arrest [29]. In addition, studies have reported that CDK4/6 signaling promotes the continuous progression and growth of cancer cell cycles, and CDK4/6 inhibitors is used as first-line or second-line treatments for breast cancer Endocrine therapy [30, 31]. In this study, we found that the depletion of CDK4 levels inhibited the proliferation, migration, and colony formation of MDA-MB-231 and BT549 cells. The downregulation of CDK4 also limited the promotion of cell proliferation, migration, and colony formation caused by overexpression of TEX19. Furthermore, classical pathway enrichment analysis and molecular interaction network analysis by IPA revealed that TEX19 may regulate the Cyclins and Cell Cycle Regulation pathways, which was confirmed by western blotting that TEX19 reduced the expression of cell cycle-related proteins CCND1 and CDK6.

In summary, TEX19 and CDK4 were significantly highly expressed in breast cancer, and high levels of TEX19 predicted a poor prognosis. TEX19 upregulated the expression of CDK4 by disrupting SKP2-mediated ubiquitination of CDK4, thereby promoting breast cancer progression. Therefore, TEX19 may be a new type of breast cancer biomarker and therapeutic target, which played a crucial role in improving the survival and prognosis of breast cancer patients. Nevertheless, we still need to further expand the clinical sample size to verify the expression a correlation of TEX19 and CDK4 in breast cancer.

### Electronic supplementary material

Below is the link to the electronic supplementary material.

Supplementary Material 1

## Data Availability

All date included in this study are available upon request by contact with the corresponding author.
