# Supplementary material for: TEX19 increases the levels of CDK4 and promotes breast cancer by disrupting SKP2-mediated CDK4 ubiquitination
Source: Cancer Cell Int. 2024 Jun 12;24:207. doi: 10.1186/s12935-024-03384-4 (PMC11170899; doi:10.1186/s12935-024-03384-4)
Supplement: Supplementary file 1 — Supplementary Material 1 [file 12935_2024_3384_MOESM1_ESM.docx]

**Supplementary figure 1 TEX19 knockdown model of breast cancer cells was successfully constructed**


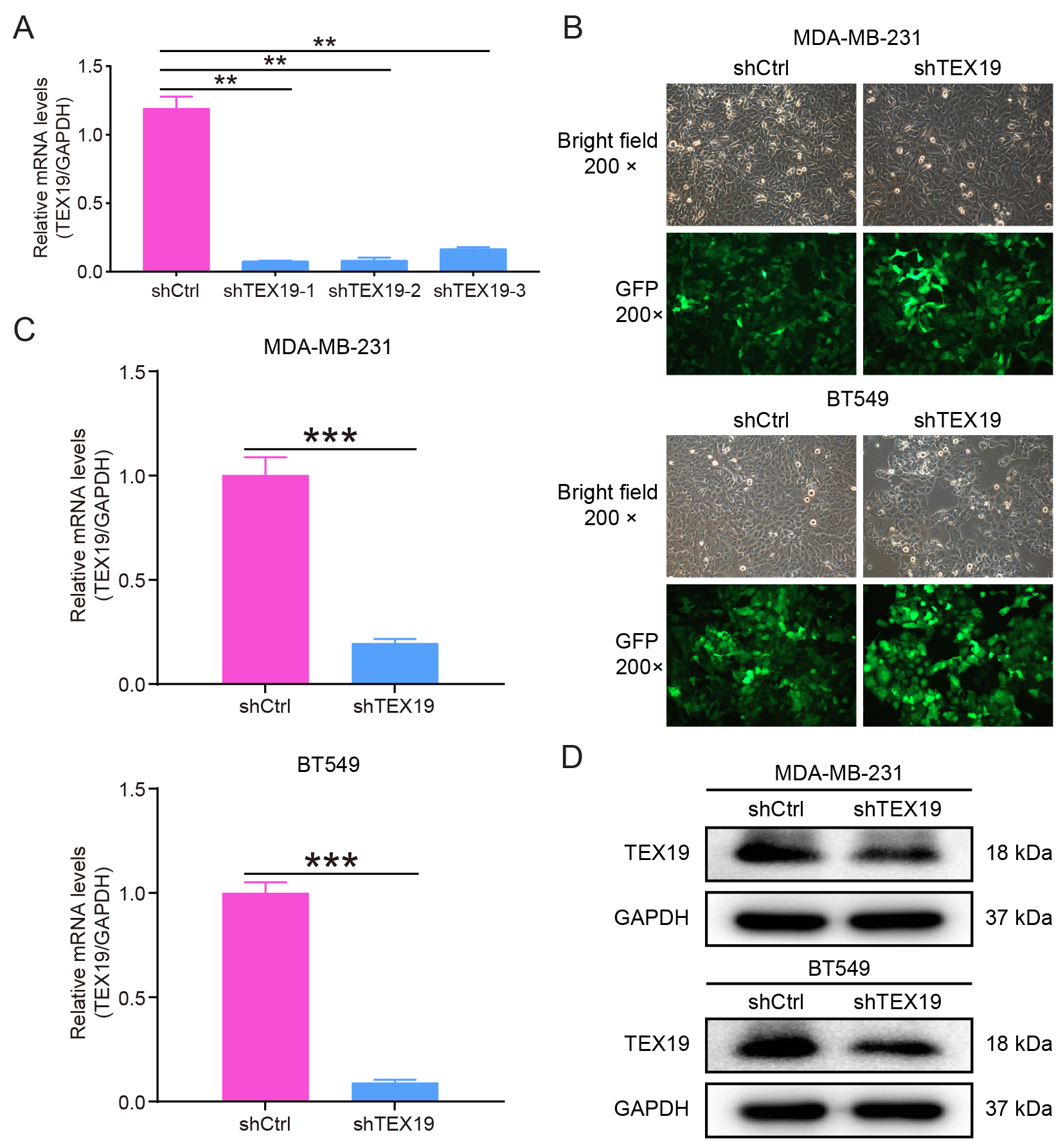


(A) Three shTEX19 lentiviruses were designed using TEX19 gene as template, and their knockdown efficiency against TEX19 was detected by qPCR. (B) The fluorescence expression of MDA-MB-231 and BT549 cells infected with shTEX19 was observed to evaluate the infection efficiency. (C) The mRNA levels of TEX19 in MDA-MB-231 and BT549 cells infected with shTEX19 was detected by qPCR to assess the knockdown efficiency. (D) The protein levels of TEX19 in MDA-MB-231 and BT549 cells infected with shTEX19 was detected by western blotting to assess the knockdown efficiency. ** P <0.01, ***P < 0.001. These cell assays were repeated for 3 times.

**Supplementary figure 2 The detection results of Human Apoptosis Antibody Array**


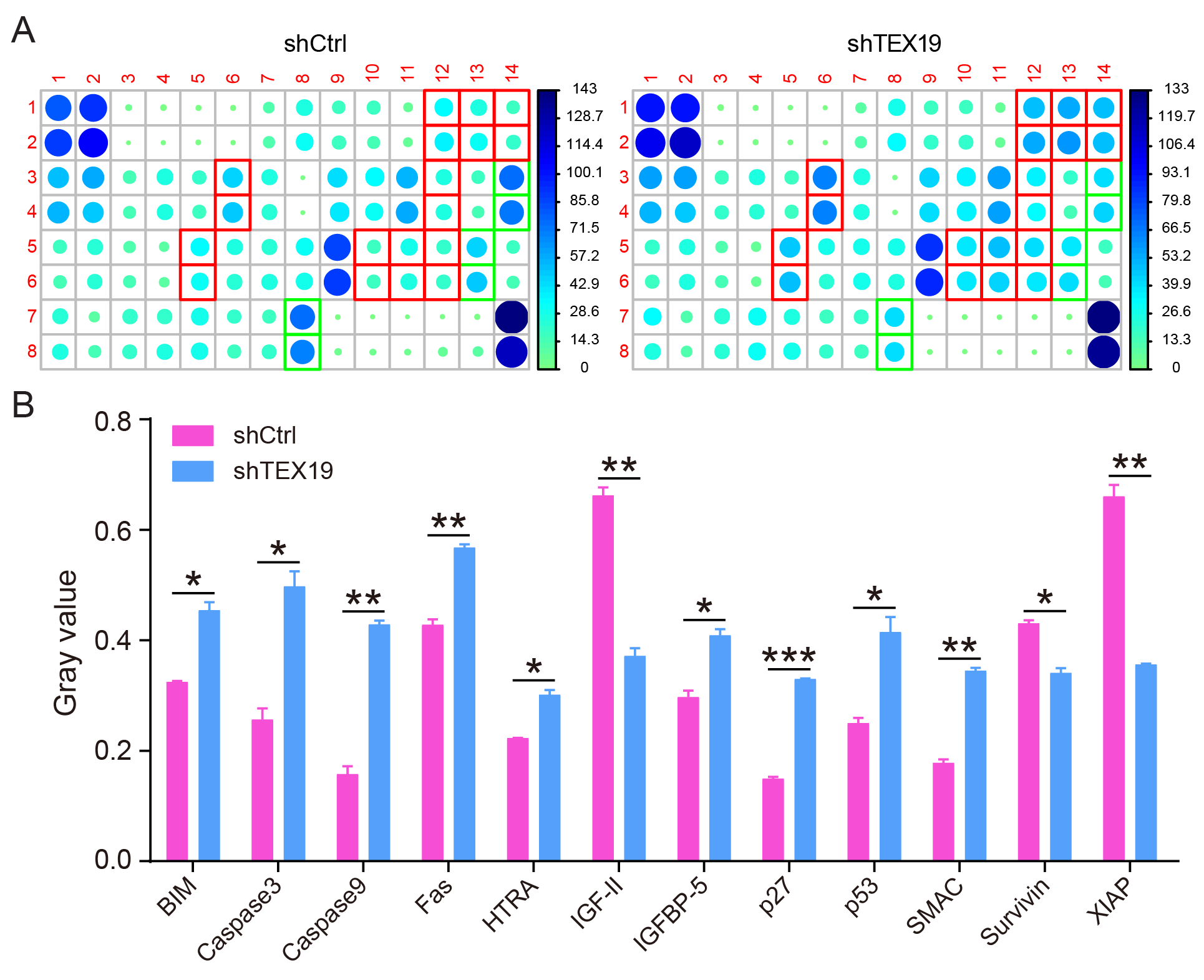


(A) The detection results of Human Apoptosis Antibody Array. (B) The histogram the measured results of several proteins with significant changes in expression levels (BIM, Caspase3, Caspase9, Fas, HTRA, IGF-II, IGFBP-5, p27, p53, SMAC, Survivin, and XIAP). *P < 0.05, ** P <0.01, ***P < 0.001.


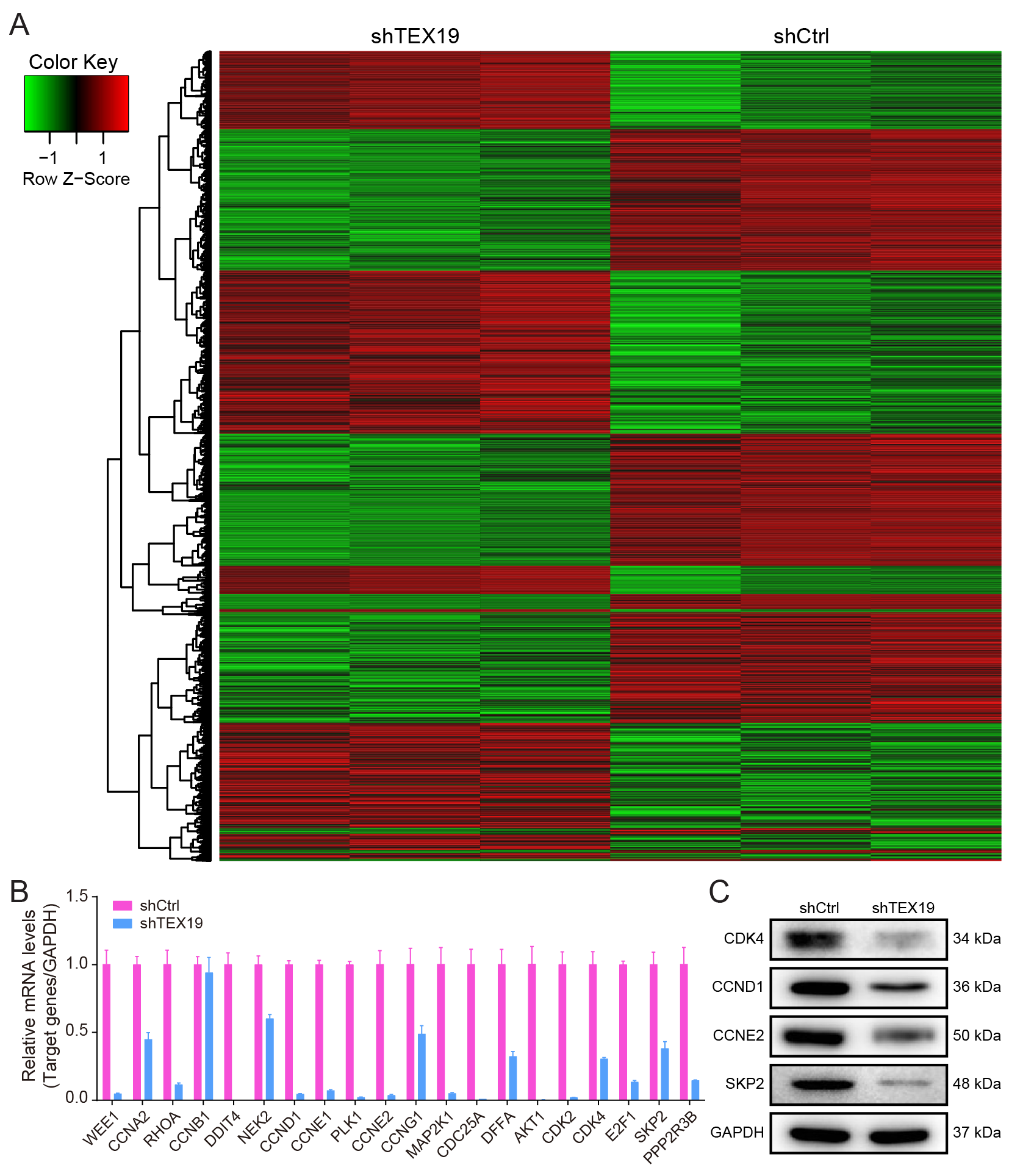


**Supplementary figure 3 The downstream molecules that TEX19 may regulate were screened**

(A) The screened differentially expressed genes were visualized in the heat map. (B) The mRNA levels of 20 significantly differentially expressed genes in TEX19 knockdown MDA-MB-231 cells were demonstrated by qPCR. (C) The protein levels of 4 significantly downregulated genes (CDK4, CCND1, CCNE2, SKP2) in TEX19 knockdown MDA-MB-231 cells were demonstrated by western blotting.


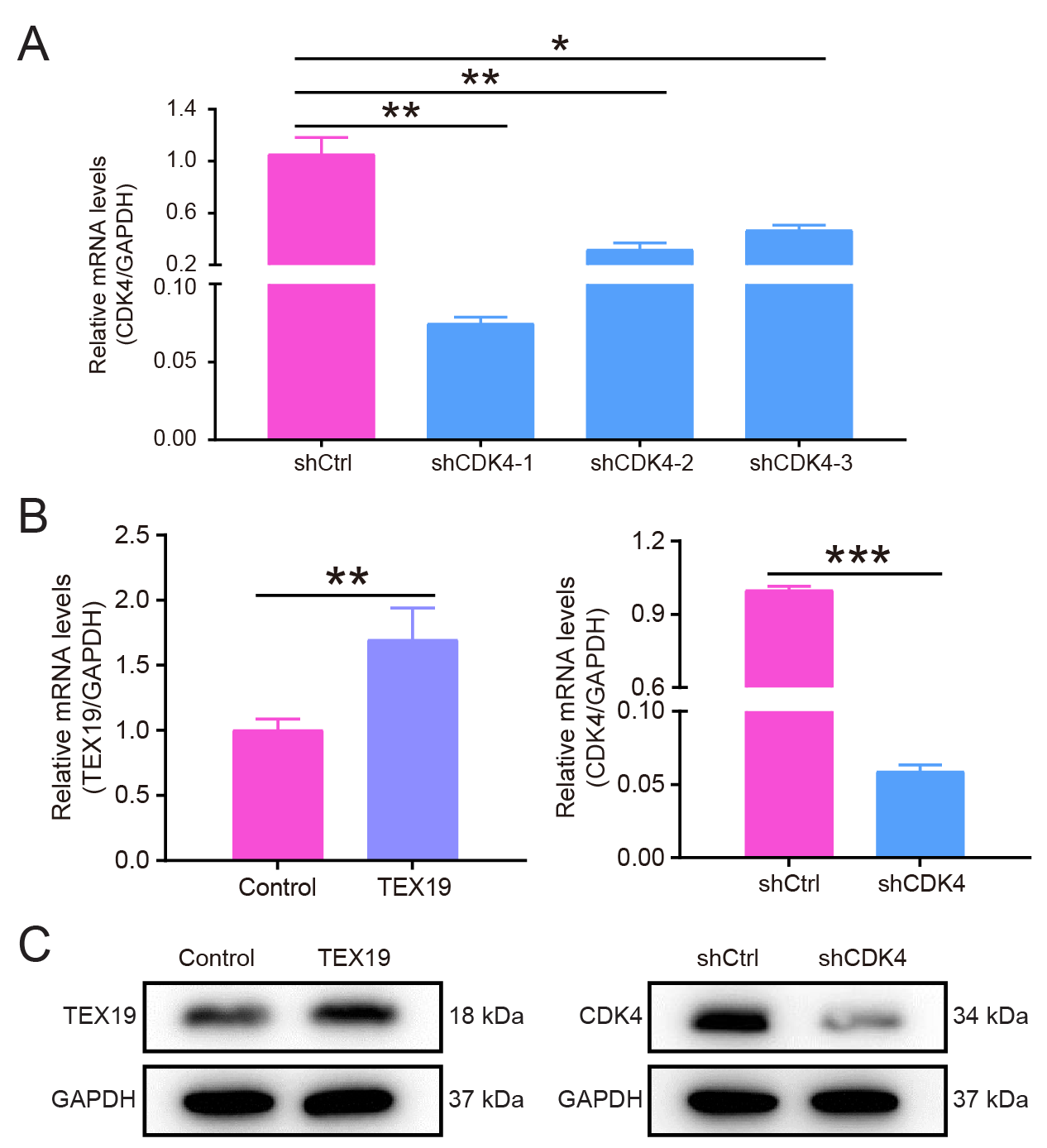


**Supplementary figure 4 Gene overexpression or knockdown model of** **MDA-MB-231 cells was successfully constructed**

(A) Three shCDK4 lentiviruses were designed using CDK4 gene as template, and their knockdown efficiency against CDK4 was detected by qPCR. (B) The mRNA levels of TEX19 and CDK4 in MDA-MB-231 cells infected with lentivirus was detected by qPCR to assess the overexpression or knockdown efficiency. (C) The protein levels of TEX19 and CDK4 in MDA-MB-231 cells infected with lentivirus was detected by western blotting to assess the overexpression or knockdown efficiency. *P < 0.05, **P < 0.01, ***P < 0.001. These cell assays were repeated for 3 times.

**Supplementary table 1.** Antibodies used in western blotting, IHC and Co-IP

| Primary antibodies | Dilution in WB/Co-IP | Source species | Company | Catalog No. |
| --- | --- | --- | --- | --- |
| TEX19 | 1:1000 | Rabbit | NOVUS | NBP2-88431 |
| CDK4 | 1:1000 | Rabbit | abcam | ab68266 |
| CCND1 | 1:1000 | Rabbit | CST | 2978 |
| CCNE2 | 1: 2000 | Rabbit | abcam | ab40890 |
| SKP2 | 1:1000 | Rabbit | abcam | ab183039 |
| DYKDDDDK Tag | 1:50/1:1000 | Rabbit | CST | 14793 |
| ERK | 1:2000 | Rabbit | CST | 4695 |
| p-ERK | 1:500 | Rabbit | CST | 4370 |
| CDK6 | 1:1000 | Rabbit | abcam | ab151247 |
| PIK3CA | 1:1000 | Rabbit | abcam | ab40776 |
| Ubiquitin | 1:2000 | Rabbit | santa cruz | sc-47721 |
| GAPDH | 1:3000 | Mouse | Proteintech | 60004-1-lg |
| Primary antibodies | Dilution in IHC | Source species | Company | Catalog No. |
| TEX19 | 1:200 | Rabbit | abcam | ab185507 |
| CDK4 | 1:100 | Rabbit | abcam | ab185507 |
| Ki67 | 1:200 | Rabbit | abcam | ab16667 |
| Secondary antibody | Dilution |  | Company | Catalog No. |
| HRP Goat Anti-Rabbit IgG (WB and Co-IP) | 1:3000 |  | Beyotime | A0208 |
| HRP Goat Anti-Mouse IgG (WB and Co-IP) | 1:3000 |  | Beyotime | A0216 |
| HRP Goat Anti-Rabbit IgG (IHC) | 1:400 |  | Abcam | ab6721 |

**Supplementary table 2.** Target sequences and shRNA sequences used for TEX19 or CDK4 gene knockdown

| Gene symbol | Target sequence |  | shRNA sequences (5'-3') |
| --- | --- | --- | --- |
| TEX19-1 | GAGATCAGCTAAGCATTTGCT | Pbr10327-a | ccggGAGATCAGCTAAGCATTTGCTctcgagAGCAAATGCTTAGCTGATCTCTTTTTG |
|  |  | Pbr10327-b | aattcaaaaaGAGATCAGCTAAGCATTTGCTctcgagAGCAAATGCTTAGCTGATCTC |
| TEX19-2 | GTCAGCATGCGGTATGAGGAA | Pbr00181-a | ccggGTCAGCATGCGGTATGAGGAActcgagTTCCTCATACCGCATGCTGACTTTTTG |
|  |  | Pbr00181-b | aattcaaaaaGTCAGCATGCGGTATGAGGAActcgagTTCCTCATACCGCATGCTGAC |
| M TEX1ISP-3 | ATGGAGATCAGCTAAGCATTT | Pbr00182-a | ccggATGGAGATCAGCTAAGCATTTctcgagAAATGCTTAGCTGATCTCCATTTTTTG |
|  |  | Pbr00182-b | aattcaaaaaATGGAGATCAGCTAAGCATTTctcgagAAATGCTTAGCTGATCTCCAT |
| CDK4-1 | CTGGTGACAAGTGGTGGAACA | Pbr10207-a | ccggCTGGTGACAAGTGGTGGAACActcgagTGTTCCACCACTTGTCACCAGTTTTTG |
|  |  | Pbr10207-b | aattcaaaaaCTGGTGACAAGTGGTGGAACActcgagTGTTCCACCACTTGTCACCAG |
| CDK4-2 | CTACATAAGGATGAAGGTAAT | Pbr10208-a | ccggCTACATAAGGATGAAGGTAATctcgagATTACCTTCATCCTTATGTAGTTTTTTG |
|  |  | Pbr10208-b | aattcaaaaaCTACATAAGGATGAAGGTAATctcgagATTACCTTCATCCTTATGTAG |
| CDK4-3 | CCGAACTGACCGGGAGATCAA | Pbr10209-a | ccggCCGAACTGACCGGGAGATCAActcgagTTGATCTCCCGGTCAGTTCGGTTTTTTG |
|  |  | Pbr10209-b | aattcaaaaaCCGAACTGACCGGGAGATCAActcgagTTGATCTCCCGGTCAGTTCGG |

**Supplementary table 3.** Primers used in RT-qPCR assay

| Gene | Forward primer sequence (5'-3') | Reverse primer sequence (5'-3') |
| --- | --- | --- |
| TEX19 | GGTGCCCACATGAACAGAGA | GGATGAAGGGGACAAGGAGC |
| WEE1 | ATTTGATGTGCGACAGACTCCT | ACTGGCTTCCATGTCTTCACC |
| CCNA2 | AGCCTGCGTTCACCATTCA | GGGCATCTTCACGCTCTATTTT |
| RHOA | TGGAAAGCAGGTAGAGTTGGC | ACATCGGTATCTGGGTAGGAGA |
| CCNB1 | AAACTTTGGTCTGGGTCGGC | TGCTGCAATTTGAGAAGGAGG |
| DDIT4 | TTAGCAGTTCTCGCTGACCG | CCAAAGGCTAGGCATGGTGA |
| NEK2 | GAAGGAATGCCACAGACGAAGT | CAAGCAGCCCAATGACCAGATA |
| CCND1 | AGCTGTGCATCTACACCGAC | GAAATCGTGCGGGGTCATTG |
| CCNE1 | CTGGATGTTGACTGCCTTGAA | CGCACCACTGATACCCTGAAA |
| PLK1 | AGAGGAGGAAAGCCCTGACT | TAACTCGGTTTCGGTGCAGG |
| CCNE2 | AACTATTTGGCTATGCTGGAGG | TTTTCAGTGCTCTTCGGTGG |
| CCNG1 | CAGAATGACTGCAAGACTAAGGG | AGTCAGTTGCCAATGGGACA |
| MAP2K1 | GGAACCAGATCATAAGGGAG | CGCTGTAGAACGCACCAT |
| CDC25A | TGGAAGTACAAAGAGGAGGAAGAG | GCCAGGGATAAAGACTGATGAAG |
| DFFA | CTAAGTTTGTGGCATTGGCTAG | CCCCGCTGTCTGTTTCATCTA |
| AKT1 | AGGAGGAGGAGGAGATGGACTT | TTGCCCAGCAGCTTCAGGTA |
| CDK2 | GGTCACATCCTGGAAGAAAGG | ATCGCAAATGCTGCACTACG |
| CDK4 | CTACCAGATGGCACTTACACCC | GCAAAGATACAGCCAACACTCC |
| E2F1 | CACTTTCGGCCCTTTTGCTC | GTGCTCTCACCGTCCTACAC |
| SKP2 | ATAGAAGTGTCCACCCTCCACG | CACCCAGAAAGGTTAAGTCGC |
| PPP2R3B | ATGACCACGCCCTTTCTACC | CCCTTCCTTCTGCACTTTTCT |
| GAPDH | TGACTTCAACAGCGACACCCA | CACCCTGTTGCTGTAGCCAAA |
